# Quantitative urban classification for malaria epidemiology in sub-Saharan Africa

**DOI:** 10.1186/1475-2875-7-34

**Published:** 2008-02-25

**Authors:** Jose G Siri, Kim A Lindblade, Daniel H Rosen, Bernard Onyango, John Vulule, Laurence Slutsker, Mark L Wilson

**Affiliations:** 1 Department of Epidemiology, University of Michigan School of Public Health, Ann Arbor, Michigan, USA; 2 Division of Parasitic Diseases, National Center for Zoonotic, Vector-Borne and Enteric Diseases, Centers for Disease Control and Prevention, Atlanta, GA, USA; 3 Centre for Vector Biology and Control Research, Kenya Medical Research Institute, Kisumu, Kenya

## Abstract

**Background:**

Although sub-Saharan Africa (SSA) is rapidly urbanizing, the terms used to classify urban ecotypes are poorly defined in the context of malaria epidemiology. Lack of clear definitions may cause misclassification error, which likely decreases the accuracy of continent-wide estimates of malaria burden, limits the generalizability of urban malaria studies, and makes identification of high-risk areas for targeted interventions within cities more difficult. Accordingly, clustering techniques were applied to a set of urbanization- and malaria-related variables in Kisumu, Kenya, to produce a quantitative classification of the urban environment for malaria research.

**Methods:**

Seven variables with a known or expected relationship with malaria in the context of urbanization were identified and measured at the census enumeration area (EA) level, using three sources: a) the results of a citywide knowledge, attitudes and practices (KAP) survey; b) a high-resolution multispectral satellite image; and c) national census data. Principal components analysis (PCA) was used to identify three factors explaining higher proportions of the combined variance than the original variables. A k-means clustering algorithm was applied to the EA-level factor scores to assign EAs to one of three categories: "urban," "peri-urban," or "semi-rural." The results were compared with classifications derived from two other approaches: a) administrative designation of urban/rural by the census or b) population density thresholds.

**Results:**

Urban zones resulting from the clustering algorithm were more geographically coherent than those delineated by population density. Clustering distributed population more evenly among zones than either of the other methods and more accurately predicted variation in other variables related to urbanization, but not used for classification.

**Conclusion:**

Effective urban malaria epidemiology and control would benefit from quantitative methods to identify and characterize urban areas. Cluster analysis techniques were used to classify Kisumu, Kenya, into levels of urbanization in a repeatable and unbiased manner, an approach that should permit more relevant comparisons among and within urban areas. To the extent that these divisions predict meaningful intra-urban differences in malaria epidemiology, they should inform targeted urban malaria interventions in cities across SSA.

## Background

The increasing urbanization of sub-Saharan Africa (SSA) may profoundly alter the epidemiology of malaria on the continent. The proportion of Africans living in cities is rapidly rising, and projected to reach 50% by 2030 [1]. Nonetheless, some debate exists over the relative importance of urban malaria. Although a number of reviews ([2-5]) assert the growing magnitude of the problem – one recent analysis estimates that urban SSA may account for 6–28% of the global malaria burden [6] – others project substantially lower figures [7]. Moreover, although malaria has been observed in cities across the continent, risk factor effects have been observed to vary significantly from one urban setting to another and, indeed, within a particular town (see, e.g., [5,8]). In large part, this reflects the heterogeneity of urban areas in Africa and the highly focal nature of malaria transmission in cities. However, it is likely that the variation in observed results among ostensibly similar areas is in part an artefact of inadequate definitions of urbanization in the context of malaria epidemiology.

In particular, the lack of well-defined terminology for urban ecotypes limits both the generalizablity of results from individual studies and the accuracy of region-wide estimates of malaria burden that incorporate prior research. The terms "urban," "peri-urban," "semi-urban" and "suburban" have no standard definition in the malaria literature, and their use without specification is likely to result in misclassification in meta-analysis and misapplication when generalizing results from prior research to new areas. Detailed and widely accepted classifications have been proposed for "slums," based on household resources and relative deprivation [9]. Similarly universal and quantitative designations would facilitate the study of urban areas as a whole. In particular, these terms should be defined in such a way that they capture ecological differences relevant to malaria epidemiology.

Beyond affecting basic understandings of urban malaria epidemiology, the lack of adequate definitions of urbanization has implications for the practice of malaria control in cities of SSA. The extreme heterogeneity of urban areas with regard to urban housing, education, household wealth, treatment access, community resources, and proximity to potential mosquito breeding sites implies that malaria interventions applied wholesale across these diverse ecological and epidemiological regimes may not achieve their goals. Rather, homogeneous intra-urban zones with consistent malaria epidemiologic profiles should be identified to permit targeting of prevention, control and treatment programs. A number of quantitative studies have explicitly considered urban-rural differences in social and behavioural factors related to malaria (e.g., [10,11]), however, only a few have specifically targeted intra-urban differences (e.g., [12,13]) or described well-defined intra-urban ecological regimes that would allow for the focusing of interventions.

In this study, quantitative clustering methods were used to identify homogenous urban units in SSA using variables expected to be related to malaria occurrence and readily available to public health officials and researchers. This approach, which was applied to urban malaria in Kisumu, Kenya, has the potential to form the basis for a more uniform process of continent-wide classification of urban areas, and a tool to focus local interventions on the areas where they are needed most.

## Methods

### Study area

Kisumu (pop. 326,407, 1999 census), on the shores of Lake Victoria in western Kenya [14], is characteristic of the demographic setting within which most SSA population growth will occur over the next 30 years [1], i.e., cities under one million inhabitants. Malaria transmission in adjacent rural areas is among the highest in East Africa [15,16]. Malaria transmission is greatest following the two rainy seasons that typically occur from April-June and October-December.

The study area was limited to the 13 administrative "sublocations" of the city (roughly equivalent to large neighbourhoods) where population densities averaged >1,000/km^2^. This threshold has been used to define urban in a review of malaria morbidity and mortality across Africa [7]. At smaller scales, population density varied considerably within the selected area. This area encompassed a range of urban ecotypes with variation in factors likely to influence malaria risk, including land use/land cover, economic and agricultural activity, distance from urban shops and health facilities, and environmental features. In this area, 202,282 people occupied 54,403 households [14] over an area of 62.3 km^2^.

### Data sources and variables

The study area was characterized at the level of the census enumeration area (EA) according to a set of variables previously reported as indicators of urbanization or of urban malaria transmission and considered accessible to local health workers. Seven variables were used: household access to electricity and to piped water [9], ownership of the dwelling [9], education level of the primary caregiver [9], distance from the city centre (see, e.g., [17]), population density (see, e.g., [18]), and normalized difference vegetation index (NDVI) (see, e.g., [19]). In particular, the first four variables constitute part of the UN-HABITAT definition for slums, while the latter three represent important elements of theoretical models of urbanization [20] that have also been observed to constitute malaria risk factors in real-world settings. Census guidelines specify that each EA should ideally comprise ~100 households, although this varied where population density or environmental features required larger or smaller boundaries to facilitate enumeration [14]. Accordingly, the classification of variables at the EA level provides a finer level of spatial detail in the places where population is most concentrated.

Variables for classification were abstracted from three sources: a knowledge, attitudes and practices (KAP) survey related to malaria covering the entire urban study area, a high-resolution multi-spectral Quickbird satellite image, and 1999 Kenya Census maps and summary data.

The sampling strategy for the KAP survey is described in more detail in a companion paper [24]. Briefly, a spatially stratified sampling scheme was used to select 4,550 sampling points corresponding to households in 473 of 567 census enumeration areas (EAs), representing an intended 10% household sample, with probability of selection proportional to population density. In this sampling strategy, each EA represented a geographic stratum, within which a sample of census-sampled structures was identified from census maps. The locations of the identified structures were cross-referenced to a GIS base map of the study area, and coordinates for the corresponding sampling points loaded into handheld GPS units. Interviewers travelled to the given coordinates and located the nearest eligible household for interview. Within each household, a resident child caregiver was interviewed with regard to their knowledge, attitudes and practices (KAP) related to malaria. A total of 4,336 interviews were completed between July 2002 and January 2003. Of assigned interview points, 95.3% yielded a valid interview. Non-response due to refusal or inability to reach an eligible respondent totalled less than 2%.

Access to electricity and piped water, household ownership and education levels were assessed in the KAP survey and summary measures (i.e., the proportion of households with access to electricity, proportion with access to piped water, proportion that owned their dwelling, and proportion where the interviewed caregiver had completed primary school) computed for each EA. Where an EA was unsampled, or where very few observations (<5) were available within an EA, the summary measure was a weighted average of proportions in the original and all adjacent EAs (in the very rare cases where this still did not yield sufficient observations, the summary measure included observations in all EAs with second-order adjacency). Household coordinates from the KAP survey were ascertained using handheld Garmin Etrex Global Positioning System units (Garmin, Olathe, KS), and distance from the city centre calculated using ArcGIS v. 9.0 (ESRI, Redlands, CA). Distance from city centre was also averaged at the EA level for classification, or averaged from adjacent EAs where few or no observations were available.

NDVI was derived from a high-resolution (2.4 m) multispectral Quickbird satellite image (DigitalGlobe, Longmont, CO) of the study area from February 2003, during the dry season. NDVI was averaged at the EA level for classification purposes. Census summary population data and census maps transferred to ArcGIS were used to compute population density at the EA level.

### Classification

Cluster analytic techniques were used to identify patterns of urbanization and classify EAs into particular ecotypes according to these patterns. Initially, a principal components analysis (PCA) was performed, using the PRINCOMP procedure in SAS v. 9.1 (SAS Institute, Cary, NC) to identify linear combinations of the original variables (i.e., factors) explaining a greater proportion of the combined variation among EAs than the original variables themselves. The EA-level factor scores obtained through PCA were used to assign each EA to one ecotype, using a k-means clustering algorithm (FASTCLUS procedure in SAS). This algorithm attempts to minimize the dissimilarity within groups through a reallocation of observations among a pre-specified number (k) of clusters. Preliminary analysis indicated the presence of three important clusters corresponding with a typical division of urban spaces into urban, peri-urban and semi-rural zones. Since the results of k-means clustering are sensitive to the initial seed, the clustering algorithm was replicated 100 times with different random seeds, and the solution chosen that maximized the cubic clustering criterion (CCC), a standard measure of fit which compares clusters identified by the algorithm to hypothetical clusters arising through sampling from a uniform distribution on a hyperbox [21].

### Validation

The geographic and demographic properties of the classification produced by the clustering method were compared with those of two alternate classifications: a) administrative designation of EAs as urban or rural by the Kenyan census [14] and b) population density thresholds (urban ≥ 1,000/km^2^; peri-urban ≥ 500/km^2^) [7]. Additionally, the distributions of three variables related to urbanization, but not used in the classification process (cooking gas use, earth floors in the dwelling, and car ownership), were characterized for each of the three classification systems (clustering administrative and population density). The extent to which each system captured variation in these factors represented a proxy for its ability to describe true variation in urbanicity.

### Human subjects

The protocol for this study was approved by the Kenya Medical Research Institute (KEMRI) National Ethical Review Committee (Nairobi, Kenya) and the Institutional Review Boards of the Centers for Disease Control and Prevention (Atlanta, GA) and the University of Michigan (Ann Arbor, MI). The research protocol and rights and responsibilities of participants were explained to potential respondents by interviewers, and written informed consent was obtained prior to all interviews. All procedures for this study were supervised by CDC/KEMRI.

## Results

**Figure 1:**
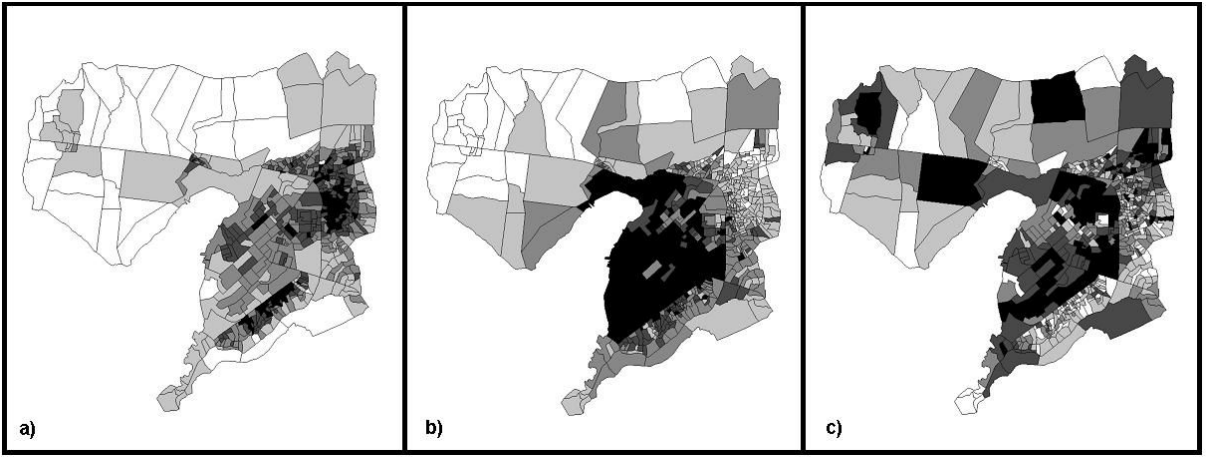
**Spatial distribution of principal component factors for urbanization-related variables**. a) Factor 1: Agriculture/Vegetation; b) Factor 2: Infrastructure; c) Factor 3: Wealth.

The PCA identified three significant factors accounting for about 70% of the variation in the original set of variables (Table 1). Factor loadings of > 0.4 were considered significant. Factor I loaded strongly on house ownership and NDVI, and had a strong negative relationship with population density. Factor II loaded strongly on access to piped water and decreased distance to the city centre. Factor III loaded strongly on completion of primary school and access to electricity. Maps of factor distributions show distinct patterns for the three factors (Figure 1).

**Table 1:** Principal Component Analysis Factor Loadings for Enumeration Area-Level Urbanization Variables

**Variable**	**Factor I**	**Factor II**	**Factor III**
Proportion of caregivers completing primary school	0.00098	-0.04665	**0.89380**
Proportion of households with access to electricity	-0.03707	0.39822	**0.72166**
Proportion of households that own their dwelling	**0.67133**	-0.16530	-0.22856
Proportion of households with piped water	0.11341	**0.86257**	0.21333
Population density	**-0.71133**	-0.17275	-0.19904
Distance from center of town	0.32208	**-0.80290**	0.00288
Mean NDVI*	**0.82191**	-0.18915	-0.01660

* NDVI = Normalized Difference Vegetation Index

**Figure 2:**
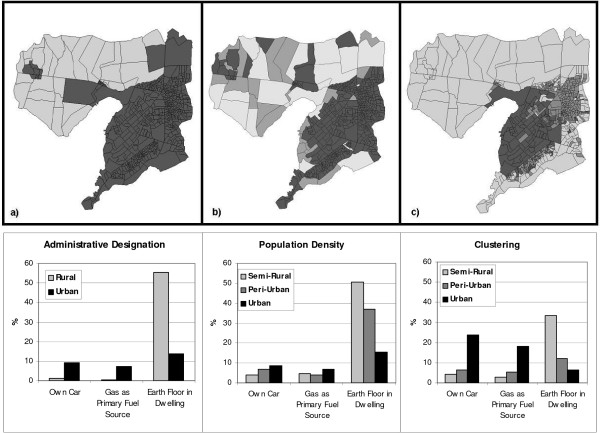
**Classification of level of urbanization in Kisumu, Kenya using three methods**. a) Classification based on administrative designation as rural or urban; b) Classification based on population density thresholds: urban > 1,000/km^2^; peri-urban > 500/km^2^; c) Classification based on k-means clustering of factors identified through principal components analysis. In all cases, darker regions are more urban.

The clustering algorithm applied to these factors indicated zones that were more continuous and geographically well defined than those identified through the use of population density thresholds (Figure 2), although administratively designated urban zones were similarly coherent. The classification produced by clustering incorporated greater proportions of the population in semi-rural (29.6%) and peri-urban (54.6%) zones than the other methods, both of which placed over 90% of people in the urban zone. The clustering classification also yielded a wider range of responses (Figure 2) and higher chi-square values (Table 2) across zones for car ownership and use of gas as a primary fuel source than the other classifications. All three classifications yielded a wide range of responses among urban zones and high chi-square values for having earthen floors in the dwelling. Unlike classification based on population density thresholds, the clustering method retained all EAs administratively classified as rural within the semi-rural category.

**Table 2:** Urbanization Indicators and Population in Relation to Three Urban Classification Systems

	**Administrative Designation**				**Population Density Thresholds**					**Clustering**				
Variable	Semi-Rural (%)	Urban (%)	X ^2^	P	Semi-Rural (%)	Peri-Urban (%)	Urban (%)	X ^2^	P	Semi-Rural (%)	Peri-Urban (%)	Urban (%)	X ^2^	P
Car ownership	1.1	9.2	29.4	**<0.01**	4.0	7.0	8.7	4.7	0.09	4.2	6.4	23.8	251.2	**<0.01**
Gas as primary fuel source	0.5	7.3	25.3	**<0.01**	4.7	3.8	6.9	3.9	0.14	2.8	5.5	18.1	178.6	**<0.01**
Earth floor in dwelling	55.3	13.9	411.3	**<0.01**	50.7	37.1	15.3	177.5	**<0.01**	33.2	12.2	6.4	322.4	**<0.01**
% of population	8.7	91.3			3.5	4.3	92.3			29.6	54.6	15.8		

## Discussion

Classification of urban areas is a non-trivial exercise in describing urban malaria. The substantially different classifications produced by the methods evaluated here illustrate the risks of generalizing or combining results from studies where urban, peri-urban and/or rural are undefined.

The clustering algorithm improves upon the other methods evaluated in several ways. First, it produces continuous zones that are both homogeneous for variables of interest and inclusive of significant portions of the urban population. These characteristics should allow researchers to focus more clearly on particular urban ecotypes, and should eventually improve the feasibility and relevance of targeted interventions for malaria control. Second, it is more strongly associated with other variables related to urbanization, and produces a wider range of responses between urban zones, than the other classification systems. The clustering approach thus appears to be both more geographically coherent and more effective at highlighting differences in urbanization-related variables across urban ecotypes than prior methods. Furthermore, it makes use of indicator variables that likely exist and are available for most SSA cities, an important consideration where resources are scarce.

Cluster analyses have been applied to a wide range of classification problems in public health and other fields [22], but apparently not for classification of urban environments related to malaria epidemiology. In this context, clustering satisfies two important theoretic goals. First, it provides a quantitative framework for cross-study and interregional comparison of urbanization-related factors, which is at best complicated and at worst misleading when terms related to urbanization are used without specification. Second, because it makes use of variables expected or shown to be related to malaria in urban areas, it represents an initial attempt to define specific urban malaria ecotypes, i.e., sets of urbanization-related factors that contribute to baseline malaria risk within a particular neighbourhood.

In fact, this study attempted to identify a minimum set of indicator variables that captured important variation in urbanization related to malaria and is generally available to researchers and public health officials or obtainable with a minimum of field effort. In doing so, three factors were observed to explain much of the variation among the original variables. While these factors,*per se*, are unmeasurable, their meanings can be inferred based on characteristic factor loadings (Table 1) and geographic distributions (Figure 1). Thus, Factor I was likely related to agriculture, or more generally vegetation, since population density is low and home ownership and NDVI high both in areas with rural character and in wealthy urban neighbourhoods. Factor II was likely a proxy for infrastructure, associated with access to piped water and proximity to the city centre. Strong loadings on use of electricity and completion of primary school may indicate that Factor III was related to wealth, although this interpretation is less straightforward.

These three elements (i.e., vegetation, infrastructure and wealth) better described urbanization related to malaria than a simple characterization based on population density in this study. Moreover, they represent three fundamental dimensions of urbanization that relate in numerous ways to malaria occurrence, risk, and outcome. For example, vegetation and infrastructure combine to determine suitability of habitats for anopheline vectors, while infrastructure and wealth both speak to the accessibility of treatment facilities. In sociology, theoretical definitions of urbanization have traditionally recognized three critical components: demographic (i.e., "increasing population size and density"), economic sector (i.e., "primarily non-agricultural labour force"), and social-psychological (i.e., "consciousness of what it means to be urban... values, attitudes, tastes and behaviours that are seen to be characteristic of urban") [20]. The factors identified here rearrange the theoretical definitions in such a way as to be relevant for malaria epidemiology. Individually, the EA-level factor scores could serve as potential risk factors in urban malaria research, while the factor maps provide insight into underlying urban variation. The cluster solution (Figure 2), which combines and makes use of all these factors, represents a nuanced picture of urban variation, captured in such a way that it relates to malaria risk.

Nonetheless, this study may have overlooked variables and combinations thereof that could better characterize "urban." Although the three factors identified here (vegetation, infrastructure and wealth) are likely to underlie any clustering solution, other variables may be more readily accessible across urban parts of SSA, and may thus prove more highly optimal for classification. Conversely, variables that would seem ideal from a theoretical standpoint (e.g., percentage of labour force involved in agriculture) may in most cases be unavailable at sufficient resolution or suitable cost to allow for their use in analysis. The search for a widely-applicable approach is vital, and should address, in addition to the optimal set of classification variables, the number of levels of classification, the most advantageous spatial scale of units, how methods can be standardized while maximizing speed and cost-efficiency, and the integration of modern tools (e.g. remote sensing, statistical methods for spatial patterns and meta-analysis).

Because only a subset of EAs (83%) in the study area was sampled due to resource limitations, environmental or socio-demographic variation in unsampled EAs might have altered some of the classifications. Similarly, the small number of samples in some EAs may have decreased the accuracy of proportions for the EA-level summary variables in those areas. Any such bias, however, should be towards the null, since unsampled EAs were randomly distributed. Another factor that may have affected the classification was the use of a dry season satellite image. To the extent that seasonal variations exist in the relative spatial distribution of vegetation, this is likely to have altered the PCA and clustering results in such a way that true malaria risk is misrepresented. Nonetheless, dry-season (February and March) NDVI measurements have been shown to be effective in describing variations in entomological and human ecological parameters in Kisumu [19]. Moreover, cloud-free remote sensing images tend to be more difficult to acquire during the rainy season. Thus, dry-season NDVI may represent the best feasible solution.

One weakness of the other methods examined here, in terms of producing an *intra-urban *classification, was the designation of zones encompassing very small populations, which would be of little use when attempting to target local interventions or identify community deficits in knowledge or malaria prevention behaviour. This effect is likely to be still more pronounced in more densely populated and/or larger urban areas of SSA. When much higher population density thresholds were applied, the resulting urbanization maps superficially resembled those obtained through clustering (results not shown), with similar population distributions among ecotypes. However, this approach identified the highest-density peri-urban slum areas as "urban" rather than the city centre or other highly urbanized zones. Population density alone is incapable of characterizing urbanization, which is a multi-factorial concept that extends beyond the most highly populated areas.

A large amount of research has gone into defining urban extents and classifying urban areas using remote sensing. Most notably, the Global Rural-Urban Mapping Project (GRUMP) has used nighttime lights (NTL) in conjunction with local lists of settled areas to construct a worldwide mask of urban extents [7]. This has been combined in various ways with the Gridded Population of the World (GPW) maps to derive estimates of urban populations. In addition, several authors have described algorithms for using remotely sensed data to classify urban areas for malaria research [23]. These efforts are essential to the construction of continental risk maps and the estimation of the overall burden of malaria attributable to urban areas, but both skirt the fundamental issue of defining urban for use in malaria epidemiology. Nighttime lights are a proxy for population density, an inadequate measure of urbanization. It is unlikely that remote sensing alone can resolve this issue; rather, it should be one component of a classification method that includes socio-demographic data.

The next step in developing this method is to verify that urban ecotypes thus defined are related to malaria. In fact, the current classification has been used to stratify KAP survey results and as a potential risk factor in case-control analyses of self-reported malaria and severe malarial anaemia in Kisumu (Siri, JS, unpublished data). Preliminary evidence from these efforts indicates that urban zones are predictive of malaria knowledge and prevention activity, as well as malaria risk.

A further step would be the application of this method to the classification of two or more similarly sized urban areas in SSA, and comparison of the resulting zones – an effort beyond the scope of the current study. Ideally, data would be pooled to produce PCA factor scores, and clustering solutions based on the joint distribution. This process would provide a framework on which to base larger-scale or continent-wide classification efforts. It is likely that classification of cities substantially different in size would yield zones that are not strictly comparable. One potential solution would be to limit comparisons to cities in defined population bands (e.g., < 1 million, 1–5 million, > 5 million). Nonetheless, the urban areas which will see the majority of population growth in the coming decades are not the very largest cities, but rather cities, like Kisumu, with less than one million inhabitants [1]. Moreover, these latter, by virtue of their lower population densities, lesser infrastructure, greater rural character, and larger "surface area to volume ratio" (and hence greater exposure to surrounding rural areas), are most likely to constitute problem areas for urban malaria.

## Conclusion

A quantitative, repeatable and unbiased process was used to classify Kisumu, Kenya into levels of urbanization related to malaria risk, based on variables that should be readily available to local health ministries and researchers alike, and incorporating both remotely-sensed and socio-demographic data. Kisumu is representative of the urban areas that will account for most of the predicted urban population growth in SSA for decades to come. Use of this classification process in urban malaria research should permit more relevant comparisons between and within urban areas, and should inform targeted urban malaria interventions, the definition and mapping of urban boundaries, characterization of ecotypes as relevant to urban malaria, and estimates of associated morbidity and mortality.

## Authors' contributions

JGS was primarily responsible for the conception and field implementation of the sampling strategy and subsequent classification process, the statistical analysis of results and the production of the manuscript draft and revisions. KAL participated extensively in study design, supervised the field project, and contributed to the statistical analysis and critical review and revision of the manuscript. BO coordinated fieldwork and provided practical input on sample implementation. DHR contributed to study design and managed data processing and verification. JV and LS played supervisory roles. MLW provided critical input on study design, statistical analysis and revision of the manuscript. All authors read and approved the final manuscript.
